# Researcher perspectives on embedding community stakeholders in T1–T2 research: A potential new model for full-spectrum translational research

**DOI:** 10.1017/cts.2019.384

**Published:** 2019-07-10

**Authors:** Sheba George, Stefanie D. Vassar, Keith Norris, Bernice Coleman, Cynthia Gonzalez, Mariko Ishimori, D’Ann Morris, Norma Mtume, Martin F. Shapiro, Anna Lucas-Wright, Arleen F. Brown

**Affiliations:** 1 Charles R Drew University of Medicine and Science, Los Angeles, CA, USA; 2 UCLA Fielding School of Public Health, Los Angeles, CA, USA; 3 UCLA Division of General Internal Medicine & Health Services Research, Los Angeles, CA, USA; 4 Olive View Olive View-UCLA Medical Center, Sylmar, CA, USA; 5 Cedars-Sinai Medical Center, Division of Rheumatology, Los Angeles, CA, USA; 6 Weill Cornell Medicine, Joan and Sanford I. Weill Department of Medicine, New York City, NY, USA

**Keywords:** Basic science, stakeholders, translational research, community engagement

## Abstract

Effective community engagement in T_3_–T_4_ research is widespread, however, similar stakeholder involvement is missing in T_1_–T_2_ research. As part of an effort to embed community stakeholders in T_1_–T_2_ research, an academic community partnered team conducted discussion groups with researchers to assess perspectives on (1) barriers/challenges to including community stakeholders in basic science, (2) skills/training required for stakeholders and researchers, and (3) potential benefits of these activities. Engaging community in basic science research was perceived as challenging but with exciting potential to incorporate “real-life” community health priorities into basic research, resulting in a new full-spectrum translational research model.

## Introduction

The National Center for Advancing Translational Sciences (NCATS) is committed to “research in the science of translation, to… (provide) the scientific foundation for improvements in translational efficiency that will accelerate the realization of interventions that improve human health”[1]; however, perspectives on how to achieve this aim have undergone substantial change. Early translational research frameworks generally depicted a linear process that starts with discovery at the basic science level (referred to as T_0_); followed by translation to humans in Phase 1 clinical trials (T_1_), then in Phase 2 and 3 clinical trials (T_2_); progressing to incorporation into healthcare practice (T_3_); and concluding with widespread dissemination to communities through population level research and policy (T_4_). Emerging frameworks suggest more complex models of iterative translation that incorporate bidirectional engagement between investigators and both clinical and community stakeholders and investigators across the translational spectrum and at multiple times [2–4].

Participation in translational research by community stakeholders – defined broadly here to include patients, families, disease advocacy groups, healthcare providers, clinical researchers, faith-based organizations, and local health departments – can help to ensure relevance and to speed up the translation of discoveries [5,6]. While strategies to engage community stakeholders in T_3_–T_4_ research have begun to make positive inroads [7–9], community participation in T_0_–T_2_ translational research is far less developed and frequently unidirectional (i.e., information transfer from scientists to communities). There remains substantial uncertainty about how to implement stakeholder engagement in early phase translation and whether stakeholder engagement can contribute positively to translational research [5,10]. Progress in engagement in early translational research will require more substantial understanding of barriers and benefits for both stakeholders and researchers and discussions between them.

As a step toward designing a comprehensive program to engage community stakeholders in T_0_– T_2_ research, our multi-institutional investigator and community partner team hosted discussion groups to better understand investigators’ perspectives on community stakeholder participation in translational science research. To the best of our knowledge, this is the first systematic attempt to assess researchers’ perspectives on both their own and community stakeholder engagement in T_0_–T_2_ research and to identify CTSI opportunities for community engagement in these under addressed translational research phases.

## Methods

### Participants

Translational science researchers at four UCLA CTSI academic partner institutions (UCLA, Cedars-Sinai Medical Center, Charles R. Drew University of Medicine and Science, and the Los Angeles Biomedical Institute at Harbor-UCLA Medical Center) were eligible for the discussion groups. A community or academic partner at each site helped identify interested researchers using snowball recruitment methods. An effort was made to include opinion leaders from each site who would be willing to participate in these types of activities. UCLA CTSI Community Engagement Research Program (CERP) sent an introductory e-mail invitation providing information about the discussion group(s) to be held at each institution.

### Group discussions

From March 2016 to January 2018, we conducted five two-hour discussion groups facilitated by a team of community members, academic faculties, and research staff. Each discussion group included a brief presentation on stakeholder participation in research and examples of engagement in T_3_–T_4_ research in Los Angeles County. Trained facilitators led semi-structured discussions with researchers on (1) potential benefits of community stakeholder inclusion in early stage translational research, (2) potential challenges/barriers, and (3) skills/training needed for both community stakeholders and investigators for successful engagement (see Supplement 1 for Moderator’s Guide).

### Data collection

Characteristics of participating researchers (career level, type of research, etc.) were collected via institutional records. Notes from each discussion group were reviewed, revised, and approved by attending community and academic team members.

### Analyses

Key themes were identified, reviewed, and revised by the community and academic team until consensus was achieved. The team developed summary lists of challenges and barriers, researcher-identified suggestions to address these barriers, and potential benefits of stakeholder engagement. Using these themes, the partnered team then identified a list of opportunities for the CTSI to support in order to incentivize stakeholder engagement in early stage research.

## Results

The five discussion groups included 37 researchers, 75% of whom were engaged in basic science studies and 25% were in clinical research; 81% of the researchers held an academic position, 11% were post-doctoral researchers, and 8% were laboratory managers (Table 1); and almost 80% had received NIH funding.

**Table 1.** Characteristics of participants (n = 37)

	*N* (%)
**Institution**	
University of California, Los Angeles	6 (16.2)
Cedars-Sinai Medical Center	9 (24.3)
Charles R. Drew University of Medicine and Science	14 (37.8)
Los Angeles Biomedical Institute at Harbor UCLA Medical Center	8 (21.6)
**Academic Role**	
Professor	16 (43.2)
Associate Professor	5 (13.5)
Assistant Professor	9 (24.3)
Post-doctoral researcher	4 (10.8)
Laboratory staff	3 (8.1)
**Holds Academic Leadership Role** (i.e., Department Chair, Division/Section Chief, Dean/Vice Dean, etc.)	14 (37.8)
Type(s) of Research*	
Basic science	28 (75.7)
Preclinical research	23 (62.2)
Clinical research	9 (24.3)
Clinical implementation	2 (5.4)
Public health	4 (10.8)
Received prior NIH funding**	29 (78.3)
**Female**	15 (40.5)
**Race/ethnicity**	
White	17 (45.9)
African American	4 (10.8)
Asian	10 (27.0)
Others	6 (16.2)

* Collecting via survey/PubMed search for survey nonrespondents. Participants were engaged in more than one category of research.

** Data abstracted from National Institutes of Health (NIH) Research Portfolio Online Reporting Tools (RePORTer).

### Benefits of Community Stakeholder Engagement

A topic mentioned prominently in most of the discussion groups was the benefit of having patients, their caregivers, and communities who are directly affected by the condition(s) being studied as part of the research team. Discussions with such patients and their caregivers were thought to increase the researchers’ own motivation for the research and could change their research priorities over time. Researchers also suggested that community stakeholders with education in other fields (e.g., accountants, teachers, etc.) have different perspectives that can be usefully applied to research (Table 2). Participating scientists indicated that improving their skill in explaining their research in lay language could enhance their ability to communicate research to funders and donors. They also observed that stakeholder engagement could contribute to the dissemination plans now required by many grant applications and would demonstrate evidence of previous collaboration between researchers and stakeholders, particularly if they had publications co-authored by community stakeholders. The researchers also felt that including stakeholders in developing dissemination strategies could expand the reach of the research to non-academic venues.

**Table 2.** Researcher-identified benefits

Benefits	CTSI Opportunities
**Researcher benefits:**	
Stakeholders ability to give input on decisions made around research goals and how they are applied and disseminated	Informed decision making on funding priorities Provide pilot funding to support and maintain partnerships
Increase ability to advocate for research that is meaningful to stakeholders and their organizations and communities	Support advocacy skills for investigators (i.e., local, regional and national policy)
Training students, and next generations of researchers to engage community stakeholders in science	Require community engagement training to all trainees in CTSA trainee programs (TL1, KL2, etc.)
Reduce skepticism in engaging stakeholders	Increase participation in community-engaged research by basic science researchers
Increase researchers’ ability to explain research in lay language to funders and policy makers	Communication training for researchers that includes lay people Provide informal opportunities for researchers to present work (i.e., Pint-of-Science, Nerd night, etc.) Provide opportunities for researchers to disseminate their research through articles in local newspapers and ethnic media, lay-language poster sessions
Discussions with patients and caregivers would lead to increased motivation for research	Development of scientific literacy training materials that are culturally and linguistically appropriate for such stakeholders
Increased ability to frame required dissemination plans for grants	Develop boiler-plate wording informed by stakeholders for use in grants and funding proposal
**Researcher-identified stakeholder benefits:**	
Community representatives’ ability to give input on decisions made around research goals and how they applied and disseminated	Community-specific training on research methods and how to communicate issues faced by their communities (i.e., Community Faculty, etc.)
Reduce mistrust of the healthcare system and research in certain communities	Increased participation in research Better health outcomes for minority communities
Increase ability to advocate for research that is meaningful to stakeholders and their organizations and communities	Support venues for researchers to disseminate and distill research for presentation to policy makers in partnership with stakeholders
Inclusion of stakeholders and community organizations in required dissemination plans for grants	Assist with grant preparation to ensure appropriate funding for stakeholders and community partners Provide pilot funding to build and maintain partnerships

Participating scientists acknowledged the history of mistrust in certain communities toward the healthcare system and biomedical research. They believed that efforts to engage communities with particular interest in the problems being studied in early stages of translational research could contribute toward reducing mistrust, increasing participation in research, and eventually enhancing health outcomes. They judged that better knowledge of the research conducted at academic institutions could lead stakeholders to advocate more effectively for communities and patients affected by the disease, which would have the potential to influence (a) the scope and direction of research, (b) advocacy for science funding, and (c) policy at local, state, and national levels, making all these activities more robust and beneficial to the interests of the community. The academicians understood that co-authored publications could also benefit stakeholder organizations by providing scholarly credentials to enhance advocacy and funding opportunities.

### Challenges of Community Engagement and Researcher Suggestions

Identification of appropriate stakeholders was the biggest concern discussed in each group. Meaningful participation in laboratory discussions would require some basic knowledge and ongoing and consistent commitment from highly motivated stakeholders (Table 3). Individuals with some background in science, or patients, families, and/or caregivers with direct experience or some familiarity with the condition being studied might be best suited to this role. However, the investigators acknowledged that many members of these groups may not have time, energy, and resources to make this additional commitment. The researchers suggested offering educational sessions for both stakeholders and researchers on how community participation in research can influence medicine and science to better address community and patient needs and enhance research quality.

**Table 3.** Researcher-identified challenges and suggestions and opportunities for the CTSI

Challenges/Barriers	Researcher-Identified Suggestions to address Barriers	CTSI Opportunities to Address Barriers and Leverage Benefits
Identification of the appropriate stakeholders	Patients with researched disease or caregivers would be ideal candidate and have greatest interest Identify opportunities to interact with stakeholders who might be interested in partnering to develop ongoing, lasting relationships	Identify and cultivate relationships with patient advocacy groups Identify opportunities for researchers to interact with stakeholders who might be interested in partnering to develop ongoing, lasting relationships Assist researchers with engaging patients in hard to research communities
Potential time requirements for researchers - Lack of time and resources to incorporate stakeholders into time-sensitive research processes	Institutional recognition/incentives for participating in stakeholder-engaged work (academic credit, promotion, vouchers or funding) Researchers need skills on how to leverage and optimize community engagement to fit within research timelines	Include requirement and training for community stakeholder involvement in CTSI-supported pilot funding Facilitate institutional change to incorporate stakeholder-engaged work in academic credit, promotion, vouchers, funding, etc.
Potential time requirements for community stakeholders – A high level of motivation and hard work would be required (may not have resources/time/energy to participate)	Offer trainings on why community engagement matters in medicine and science	Develop training for both researchers and stakeholders on value of community engagement in medicine and science Offer stipends for stakeholder participation
Level of technicality and specificity in research meetings may pose a barrier for stakeholders	Community stakeholders need basic scientific vocabulary and knowledge needed Laboratory-specific information that is more accessible can be prepared by graduate students and fellows	Support stakeholders participation in Mini-medical school training programs Hold training on general laboratory safety for stakeholders
Building trust between researchers and stakeholders – Community is mistrusting of researchers due to unethical research done in the past	Stakeholders need trainings to have confidence and ability to question scientists about their work. Researchers need skills in communicating their science in lay language to stakeholders Partner Priorities need to be communicated and understood early by both researchers and community partners	Develop Team Science and Communication Modules for both stakeholders and academic partners Support pilot funding for building partnerships
Administrative and logistical barriers: Lengthy institutional clearance process to allow stakeholders on campus/in laboratories Parking and travel for stakeholders	Begin stakeholder clearance process early and can mimic processes used for donors Hold meetings outside laboratory setting	Assist with security and human research clearances (HIPAA, CITI etc.) Provide stipends for stakeholder parking and travel

The level of technical discourse in research meetings may pose a barrier to stakeholder comprehension of the discussion and may intimidate them from participating in it. All researchers agreed that stakeholders would need to complete two kinds of trainings: (1) general training for research (e.g., general laboratory safety for those embedded in laboratories and human subject research certification) and (2) training specific to the work of the research group (basic scientific theory and methods and laboratory-specific knowledge about animal models, laboratory techniques, common acronyms, etc.). Some felt that graduate students and fellows may be well suited to lead laboratory-specific information sessions directed to community stakeholders. Researchers acknowledged that it will be important to explain to stakeholders that failure or null results are frequently part of the process and may promote new discoveries.

Researchers who had worked previously with community stakeholders noted that building trust between academic and stakeholder partners requires time and commitment from both partners. They also endorsed the need for a discussion of the distinct priorities of researchers and community stakeholders early in the partnership and at regular intervals, as priorities may change over time. For example, academic faculty members are often overwhelmed by multiple projects and priorities, and it may be difficult to consistently dedicate the time for meaningful community engagement if it is not a requirement of a funded grant. Additionally, embedding stakeholders in laboratory research may require frequent discussions about common laboratory procedures (especially early in the partnership) that may slow down time-sensitive research processes.

Logistical and administrative barriers to community stakeholder participation were described at all sites: the need for credentials to enter the laboratory or building (which may require background checks), parking, and travel time, and reimbursement. Most researchers felt that these were not overwhelming obstacles but could be time consuming and lengthy processes, especially to get security clearances. Several observed that allowances are made for donors to visit laboratories, and their institutions could employ a similar process for stakeholders. Others suggested an option of holding regular meetings with stakeholders outside the laboratory setting, or even outside the academic setting (i.e., at a community venue or library).

## Discussion

We conducted one of the first extensive assessments of potential barriers and facilitators to inculcating community engagement into basic and preclinical translational research. We found overall broad support for such approaches, but also elicited key needs to effectively conduct community–basic science partnerships. Our findings are consistent with the reports examining challenges for basic scientists to partner with clinical investigators. These challenges include complex regulatory requirements and limited recognition and funding for translational research [11], barriers to engagement such as identifying funding sources, preparing a budget for a grant application, and establishing collaborations and consultant agreements along with limited infrastructural support for establishing partnerships [12]. Our findings also support the emerging use of novel structured approaches to community engagement such as community engagement studios, which provide a supportive space for researchers to engage with stakeholders [13].

Other challenges that have been identified include bridging the gap between the structures, processes, and goals of research institutions, healthcare organizations, and community stakeholders in order to facilitate successful translational research (5). Such gaps include infrastructural challenges such as the lack of a collaborative institutional environment, and the increased bureaucratization of universities leading to an audit culture in research [14]. For example, to provide legal protection, research institutions may require multistep review processes taking many months before community stakeholders can be brought into translational science environments. Similarly, if the institutional culture for collaboration is weak, effective partnering efforts engaging community stakeholders in translational research may be difficult.

A more bidirectional and collaborative approach to translational research requires improved understanding of several potential issues, including (1) how to meaningfully identify and involve communities from the earliest stages of research so that they can participate in establishing research agendas and priorities, study designs and the whole innovation trajectory [9,15–17]; (2) how to change the culture of research at the institutional level to eliminate “siloed” working environments and undue regulatory and bureaucratic burdens that may preclude engaging community stakeholders [18]; (3) how to achieve bidirectional and iterative interaction between community and researchers [19,20]; and (4) what types of additional skills do basic scientists and community stakeholders need to engage together. Based on the discussion themes and researcher suggestions, we identified several opportunities at the UCLA CTSI level that support such an approach to address challenges and opportunities to community stakeholder engagement in basic science research (Tables 2 and 3).

Our study has some limitations. Because these findings are based on a convenience sample of researchers, they may not be generalizable to all basic scientists. Additionally, our team included several experienced community stakeholders whose familiarity with research may have mitigated concerns and apprehensions of basic scientists during the discussion groups. A parallel study to explore community stakeholders’ perspectives on engaging in basic science research is a necessary complement to this study. We made an effort to seek out opinion leaders; therefore, nearly 40% of the participants were in leadership positions, and thus the sample may not represent all faculty in T_1_–T_2_ research.

In summary, engaging community stakeholders in basic science research was perceived by investigators as challenging, but with exciting potential to incorporate “real-life” community health priorities into basic research, resulting in a new model of full-spectrum translational research. Realizing this potential will require that such stakeholders receive appropriate scientific literacy training and that participating scientists enhance their communication skills in translating the content and relevance of their science into lay language. Finally, while the scientists participating in our discussion groups did not identify this need, researchers may also need education on the sociocultural contexts, needs, and experiences of different types of community stakeholders for both individuals (e.g., patients and community activists) and groups (e.g., patient advocacy and community organizations).

## Supplementary material

For supplementary material accompanying this paper visit https://doi.org/10.1017/cts.2019.384. click here to view supplementary material
