# Researcher perspectives on embedding community stakeholders in T1-T2 research: A potential new model for full-spectrum translational research – ADDENDUM

**DOI:** 10.1017/cts.2019.399

**Published:** 2019-10-02

**Authors:** Sheba George, Stefanie D. Vassar, Keith Norris, Bernice Coleman, Cynthia Gonzalez, Mariko Ishimori, D’Ann Morris, Norma Mtume, Martin F. Shapiro, Anna Lucas-Wright, Arleen F. Brown

In the original publication of this article, author Bernice Coleman’s affiliation was missing. The correct affiliation of Dr. Coleman is Cedars-Sinai Medical Center, Nursing Research Department, Los Angeles, CA, USA.

The article has since been corrected.
