# Supplementary material for: Researcher perspectives on embedding community stakeholders in T1–T2 research: A potential new model for full-spectrum translational research
Source: J Clin Transl Sci. 2019 Jul 10;3(2-3):120–4. doi: 10.1017/cts.2019.384 (PMC6802407; doi:10.1017/cts.2019.384)
Supplement: Supplementary file 1 [file S2059866119003844sup001.pdf]

UCLA CTSI Community Engagement & Research Program
Embedding Stakeholders in Basic Science Research
Moderator and Discussion Guide

1. Introductions
2. Slides introducing project
3. Why engage community in basic research? Can questions from patient/community stakeholders unfamiliar with research spark new ideas?
    - Examples of specific experiences that worked

4. What are areas of research that would be beneficial to community and patient/community stakeholders?
    - Are some areas of research more amenable to community engagement?

5. What are possible challenges to implementing such a program?
    - What skills and training are needed by community stakeholders and researchers?
    - What skills and training are needed by researchers to communicate their work effectively to community and patient stakeholders?
        - "To sell your questions, you have to listen to the questions of others to find common questions, common ground "

6. What would such a community stakeholder embedded team look like? How might it best work?
7. How would you define success for this program from the perspective of different stakeholders (Community stakeholders, Junior vs. senior researchers/ the University, Patients, Policy makers)
8. If successful, how might we sustain this program?
    - How do you keep both researchers and community stakeholders engaged for the long term?
